# How the Knowledge of Interactions between Meningococcus and the Human Immune System Has Been Used to Prepare Effective *Neisseria meningitidis* Vaccines

**DOI:** 10.1155/2015/189153

**Published:** 2015-08-17

**Authors:** R. Gasparini, D. Panatto, N. L. Bragazzi, P. L. Lai, A. Bechini, M. Levi, P. Durando, D. Amicizia

**Affiliations:** ^1^Department of Health Sciences, University of Genoa, Via Pastore 1, 16132 Genoa, Italy; ^2^Department of Health Sciences, University of Florence, Viale G.B. Morgagni 48, 50134 Florence, Italy

## Abstract

In the last decades, tremendous advancement in dissecting the mechanisms of pathogenicity of *Neisseria meningitidis* at a molecular level has been achieved, exploiting converging approaches of different disciplines, ranging from pathology to microbiology, immunology, and omics sciences (such as genomics and proteomics). Here, we review the molecular biology of the infectious agent and, in particular, its interactions with the immune system, focusing on both the innate and the adaptive responses. Meningococci exploit different mechanisms and complex machineries in order to subvert the immune system and to avoid being killed. Capsular polysaccharide and lipooligosaccharide glycan composition, in particular, play a major role in circumventing immune response. The understanding of these mechanisms has opened new horizons in the field of vaccinology. Nowadays different licensed meningococcal vaccines are available and used: conjugate meningococcal C vaccines, tetravalent conjugate vaccines, an affordable conjugate vaccine against the *N. menigitidis* serogroup A, and universal vaccines based on multiple antigens each one with a different and peculiar function against meningococcal group B strains.

## 1. Introduction

The immune system protects humans from attack by microorganisms such as bacteria, viruses, protozoa, fungi, parasites, and organisms such as helminths. The skin is the first barrier and its protective action is enhanced by bodily secretions, such as sweat and sebum, which exert a broad antimicrobial activity [1, 2]. The mucous membranes are protected by external and internal secretions, such as tears, saliva, and mucus, which contain molecules that can neutralize bacteria. Tissues such as the skin and mucous membranes are populated by immune cells, which can act against the microorganisms that circumvent the first physical and biochemical barriers.

The immune system is very complex and its defensive response is subdivided into innate and adaptive responses [3]. The innate response triggers an immediate, nonspecific, general action and is activated by typical signs of infection. The adaptive response is able to develop a highly specific, extremely accurate action, which is stored in the so-called immune memory.

This paper provides an overview of the interaction between the immune system and Gram-negative bacteria with particular reference to* Neisseria meningitidis* in the perspective of developing new vaccines against this pathogen.

## 2. Gram-Negative Bacteria and Immunity

### 2.1. Outer Membrane Components

Over thousands of years, bacteria have developed several mechanisms whereby they can circumvent the immune system. Specifically, Gram-negative bacteria possess a complex of envelopes, which allow the selective passage of nutrients into the cell and the excretion of metabolic waste outside. Structurally, Gram-negative bacteria possess an outer membrane (OM), which, together with the peptidoglycan and inner membrane (IM), delimits the periplasm and cytoplasm compartments. Many molecules of glycolipids, especially lipopolysaccharide (LPS), emerge from the outer leaflet of the OM, while, from the inner layer of the OM, lipoproteins reach the peptidoglycan, with which they engage. Moreover, proteins such as porins cross the OM; these are very important for the active, passive, and selective permeability of small molecules, ions, and water [4]. Most porins have a trimeric structure and an oval shape. The bacterial porins perform many functions; indeed, they help the microorganism to adhere to the cells of the host tissue and to evade the defence mechanisms of the human body, thereby favouring invasion of the host. They are also able to elicit both innate and adaptive immunity. Porins can inhibit phagocytic activity [5] and activate the complement system by means of both classic and alternative pathways [6]. For instance, Neisserial porins can activate the transport of NF-*κ*B into the nucleus of B and dendritic cells (DCs) [7]. The DNA/NF-*κ*B complex then recalls other proteins, such as coactivators and RNA polymerase, which transcribe the DNA into mRNA; finally, this mRNA is exported to the cytosol and translated into proteins. This leads to a change in the function of the cell; for example, the cell may begin to produce proinflammatory cytokines.

Porins are clearly involved in the induction of proinflammatory activity, although it is not known which toll-like receptors recognize them. By contrast, it is known that LPS stimulates toll-like receptors 2 and 4 [8]. Three distinct regions characterize LPS, namely, lipid A, which fixes the molecule to the outer leaflet of the OM, the core polysaccharide which binds to lipid A by means of a disaccharide phosphate bridge, and antigen O, which is the most distal portion. The general structure of LPS is fully conserved, while the core oligosaccharide is highly variable.

Toll-like receptors are a family of conserved signal transducers able to induce an innate immune response. To date, at least 11 mammalian TLRs have been identified. Their stimulation by bacterial components activates the innate immune response. TLR2 recognizes peptidoglycan, lipopeptides, and bacterial proteins. However, it is interesting that LPS can overstimulate the innate immune response, thereby eliciting inflammation. As a result, the normal defences may not function correctly. Furthermore, it should be borne in mind that TLR5 recognizes flagellin, which is the main component of bacterial flagella [9]. For example, mutations of the TLR4 gene contribute to development of severe meningococcal infections [10]. In addition, through the recognition of* N. meningitidis* DNA, TLR9 exerts strong protection against the microorganism [11].

### 2.2. Innate and Adaptive Immune Responses

The innate immune system is able to detect other conserved microbial components, called pathogen-associated molecular patterns (PAMPs), such as nucleic acid structures, lipoteichoic acid, and peptidoglycan [12]. The pattern recognition receptors (PRRs) of immune cells include, in addition to TLRs, the NOD-like receptors (NLRs) and the RIG-1-like receptors (RLRs), which are able to recognize microbial components in the cytosol [13]. TLRs, NLRs, and RLRs are able to activate mitogen-activated protein kinase (MAPK) and the transcription of NF-*κ*B factor. A different set of NLRs helps to activate caspase-1 and the consequent assembly of inflammasomes [14].

The granulocytes and macrophages are the first cells that participate in the activation of the innate immune response. Shortly afterwards, the DCs and natural killer cells are activated. Specifically, neutrophils produce antimicrobial proteins, such as LL37, alpha and beta defensins, enzymes [15], interferons (IFN) alpha, beta, and gamma, C-reactive protein, and chemokines, contribute to activating the complement cascade. Macrophages produce reactive oxygen species (ROS) (e.g., H_2_O_2_) and reactive nitrogen species (RNS). Subsequently, DCs, which can also be activated by TLR2 and TLR4, activate natural killer (NKs) cells [16] and induce maturation of CD4+ T cells [17–22].

Many bacterial components are able to stimulate the adaptive human immune response. Porins can activate the translation of NF-*κ*B in the nucleus of B and DCs, while class I Pilin E induces highly specific antibodies (Abs) and class II induces cross-reacting Abs. Furthermore, complement cascade activation, as well as particularly C3b activation, opsonizes antigens, thereby enabling APCs to activate the adaptive response.

## 3. *Neisseria meningitidis* and Immunity

### 3.1. Meningococcal Genome

Meningococci have developed several “immunoescape” strategies [23], the molecular bases of which can be understood by taking into account the nature of the Neisserial genome. Progress in the field of molecular biology and the introduction of high-throughput technologies (HTTs) have tremendously advanced our understanding of the complexity of the Neisserial machinery. By using sophisticated approaches such as whole-genome sequencing (WGS) and microarrays, functional genomics investigations have uncovered the mechanisms that facilitate or hinder* N. meningitidis* growth, colonization, and invasion and have helped to explain its extraordinary intrastrain variation and adaptation to the environment. Other techniques, such as genome-wide association studies (GWAS), have shed light on the pathogen-host interaction and the host's susceptibility to the microbe. Genomics and postgenomics have not only increased our knowledge of the biology and pathogenesis of* N. meningitidis* but proved to be extremely useful in discovering candidate antigens and in developing effective new vaccines [24].

Being a naturally competent pathogen,* N. meningitidis* has a highly dynamic, plastic, and flexible genome with a size range of only more than 2,000 kilobases [25]. This genome differs from other microbial genomes in that it lacks some of the typical two-component systems and sigma factors [26]. Despite being relatively small and compact, it has elaborated a variety of mechanisms that contribute to explaining its high adaptability both to host and to environment. Meningococcus is usually polyploid, containing up to 2–5 genomes—polyploidy being a sign of virulence—while* N. lactamica* is monoploid [27]. Neisserial pathogenicity is intrinsically polygenic [28] and is given by a variety of different pathogenic islands (PAIs or genomic islands, GEIs), including gonococcal genetic islands (GGIs) [29] and a recently discovered meningococcal disease-associated (MDA) island [30].

The nature of the Neisserial chromosome and the presence of extrachromosomal material contribute to explaining an important immunoescape strategy, known as structural or antigenic variation, which consists of camouflage of the Neisserial repertoire expressed. Basically, it can involve horizontal or lateral gene transfer (HGT/LGT) (mainly via transformation and, to a lesser extent, via conjugation and phage transduction) and allelic exchange/rearrangement of genes or gene portions taken up from the environment (Table 1). In addition, as its genome contains multiple copies of certain genes, for example, opacity factor proteins and pilins [31], homologous intragenic recombination also results in frequent surface structural variation.

Moreover, the pathogen hosts a number of prophages, from the Mu-related family to the phage l-related group and the family of filamentous M13-like phages [27, 32]. The most widely studied sites for phage integration are known as duplicated repeat sequence 3 (dRS3) [26], which belong to the family of Neisserial intergenic mosaic elements (NIMEs). Plasmids, such as pJS-A and pJS-B, also play an important role [33].

Another surface modulation occurs via phase variation, a process involving the modulation of gene expression via a mechanism of on/off switching (transition from an expressed state of the gene to an unexpressed one or vice versa). Besides this kind of “functional” phase variation,* Neisseria* can also undergo a “structural” switch, namely, a transition between two forms of a gene product. The genes, which are involved in this strategy, are termed “contingency genes” [26] and can be coupled and interlinked in structures called phasevarions (phase-variable regulons) [34], which have a regulatory function. Phase variation includes a variety of sophisticated mechanisms [35], such as slipped strand mispairing (SSM) [36], microsatellite instability [37], and reversible insertion of minimal mobile elements (MMEs) [38]. Therefore, these mechanisms can involve single nucleotides (homopolymeric repeats) or complex nucleotidic structures (short tandem repeats), occurring either upstream of a gene in the promoter or within an open reading frame (ORF)/coding sequence (CDS). Changes upstream of a gene result in modulation of its transcriptional efficiency and therefore of its final protein concentration. This is, for example, the case of Opc, porin A (*por*A), and* fet*A genes. Alterations within a gene, which insert* de novo* stop codons, alter the full translation of the gene. An example of this mechanism is provided by the opa genes and the genes coding for adhesins, such as* nad*A [39]. Phase variation of opa genes has been extensively characterized: they occur in four distinct copies and code for similar, but not identical, proteins. Phase variation can thus involve one copy or another, independently of each other, and can result in eleven variants. In this case, phase variation is therefore equivalent to antigenic variation. Besides opacity factor proteins, phase variation can involve up to hundreds of genes [40]: from the genes coding for pilins [41] or for proteins involved in genome maintenance and DNA repair [42, 43] to genes encoding proteins involved in the cell cycle control and regulation [44], autotransporters [45, 46], or enzymes like the pilin phosphorylcholine transferase* ppt*A [47] or the glycosylase* mut*Y [48], among others [49, 50]; the reader is referred to Table 2, which provides a more detailed overview of the phase-variable genes. Moreover, new mechanisms leading to phase variations have been discovered [51].

The mechanism implying MMEs involves different kinds of genetic elements, such as the Correia repeats (CRs) and the Correia repeat-enclosed elements (CREEs), known also as the* Neisseria* miniature insertion sequences (NEMIS) [52, 53], which constitute about 2% of the Neisserial genome [54]. Other genetic elements are the insertion sequence (IS) elements, such as IS1016-like, IS1106, IS1301 [55, 56], and IS1655 [57].

It is worth noting that the number of genes involved in phase variation is enormously greater than for any other pathogen studies so far [58]. Some genes are “phasotypes”; that is to say, they play a role in carriage and are downregulated, favouring host persistence [59].

As already mentioned, in some cases antigenic/structural variation and phase variation, albeit conceptually two distinct mechanisms, cooperate in increasing the genetic complexity of the Neisserial genome. Antigenic variation of LPS, for example, can derive from phase variation of one or more enzymes involved in the synthesis of the oligosaccharide chain by SSM, or by modification of LPS, for example, by glycosylation [60–62], sialylation [63, 64], or acetylation [65, 66], which, moreover, confer resistance to neutrophil-mediated killing.

Thus, both antigenic and phase variations concur in enabling* Neisseria* to evade the immune system [26, 27].

### 3.2. Meningococcal Capsule

LPS and themeningococcal capsule (CP) are the two major virulence factors of* N. meningitidis*. Specifically, the capsule displays a large variability of surface antigens, on the basis of which 13 different* N. meningitidis* serogroups have been identified. The CP contributes in an important way to the camouflage of the microorganism, which thus can better circumvent the immune system's defences. The clearest expression of this phenomenon is given by the molecular mimicry [67]. This can be seen in the nature of the polysaccharide CP of serogroup B meningococcus, a homopolymer of *α*2-8-linked sialic acid, which is identical to a neural cell adhesion molecule, NCAM-1 [68]. Moreover, lacto-N-neotetraose (L-NNT) in the lipopolysaccharide of virulent strains is similar to an antigen expressed on red blood cells [69–73]. Further mechanisms of molecular mimicry have been recently discovered and described [74].

During the first phase of infection, meningococcus has to avoid the surface defences of the nasopharynx, such as the peptides secreted at the mucosal surface [90] and IgA secretory Abs [90, 196]. To this end, the meningococcus can aggregate into clusters and produce abundant OM vesicles (OMVs), thus managing to hide its surface antigens and to deflect the action of the surface defences from the bacterial cell [196]. In addition, the CP protects* Neisseria* from cationic antimicrobial peptides (CAMPs), including cathelicidin [196]. Conversely, the presence of capsular polysaccharide restrains the invasion and colonization of the nasopharyngeal barrier by hiding the adhesins and invasins of the meningococcus [143, 158]. On the other hand, the presence of the capsule may allow the microorganism to pass unharmed through the intracellular environment by exploiting the system of cell microtubules, at least in the case of serogroup B* N. meningitidis* [197]. Moreover, the CP is essential for meningococcal growth and survival in the bloodstream and cerebrospinal fluid, increasing serum resistance. During the different stages of infection, the capsule may hinder or promote the survival of* N. meningitidis* in the human host; indeed, the microorganism can modulate the production of capsule components, which depends on 4 genes, three of which—*sia*A,* sia*B, and* sia*C—are situated in the* cps* locus. The* sia*D gene induces the production of polysialyltransferase, which allows the polymerization of sialic acid. For instance, in the early stages of infection, the production and assembly of sialic acid are downregulated [198]. Another example of polysialyltransferase system is given by* oat*WY [178].

In addition to the above-mentioned actions, the most important virulence activity of the CP is probably the significant impairment of both Neisserial adherence to DCs and the phagocytic killing of bacteria; indeed, the CP inhibits both the opsonic and the nonopsonic phagocytosis of* N. meningitidis* [199]. It prevents the formations of effective Abs against* N. meningitidis*.

CP downregulates both classical and alternative complement pathways and prevents the proper insertion of the membrane attack complex (MAC) [200, 201]. LPS also contributes to inhibiting MAC deposition [201, 202].

Moreover, CP switching contributes to escaping detection and killing. This is a complex phenomenon due to microevolution and usually involves Neisserial strains expressing sialic acid (e.g., the shift from serogroup B to C, from serogroup C to W-135, from serogroup Y to W-135, and from serogroup Y to B) [79–82, 203]. The molecular basis is provided by the allelic replacement of the sialic acid CP polymerase.

Surprisingly, nanostructured materials such as multiwalled carbon nanotubes (MWNTs) and mesoporous silica have been found to increase* Neisseria*'s transformational capacity [83, 84].

### 3.3. Major and Minor Adhesion Mechanisms of* N. meningitidis*

*N. meningitidis* possesses a multifaceted system of adhesins, such as pili and other systems of adhesion (i.e., opacity-associated proteins OpA and OpC). Adhesion is probably a coordinated process mediated first by pili, which are composed of several proteins; the most important of these is Pilin E (PilE) [204], but Pilin C (PilC) [205] and the secretin Pilin Q (PilQ) [206] have also been described. PilE is the main constituent of the* Neisseria* type IV pilus. In 1984, Diaz et al. identified proteins I and II as the main components of the type IV pili and noted that Abs against protein I were highly specific [207]. Subsequently, Pilin E was classified as belonging to class I and class II. Class I Pilin E is highly variable, while class II Pilin E is highly conserved [208, 209]. For this reason, class II Pilin E has been suggested as a candidate antigen for a vaccine against meningococci [147]. The regulation of pilin genes is quite complex [97, 115, 116, 190].

Other components are as follows:* pil*V [210],* pil*P,* pil*D (a prepilin-processing leader peptidase),* pil*F and* pil*T (traffic NTPases),* pil*G (involved in the pilus assembly),* pil*M (pilus biogenesis protein), and* pil*W (involved in the pilus stabilization) [148], among others.

Although the interactions between type IV pili and cellular receptors are not completely known, they may interact with a membrane cofactor protein, named CD46 receptor, and with alpha 1 and alpha 2 integrins [211]. However, it is known that pili contribute to the aggregation of Neisserial cells [212]. This fact, associated with the ability of pili to act as a signalling protein by interacting directly with the *β*2-adrenergic receptor, contributes to the depletion of junction proteins, thus helping meningococcus to pass through the epithelial and endothelial cells and, subsequently, to cross the blood-brain barrier (BBB) [75, 213].

Although pili are essential to the first phase of Neisserial adhesion to the cells of the airways, other adhesion molecules, such as LPS and porin A, intervene to strengthen the microbial bond. In particular, OpcA and OpcC appear to be very important; indeed, OpcA binds carcinoembryonic antigen cell adhesion molecules (CEACAMs), heparan sulphate proteoglycan (HSPG) and integrins [179–183, 214]. Opc proteins can combine with HSPGs and, through vitronectin and fibronectin, with their integrin targets. Furthermore, Opa proteins are able to elicit innate human defences that favour the survival of* N. meningitidis*, while actively suppressing adaptive immune responses that would eliminate the pathogen [184]. The variability of the expression of different Opa proteins could play a major role in prolonging the state of infection by circumventing the humoral host immune response [215].

The adhesion and penetration protein (*app*) [103], which is a member of the autotransported family of secreted proteins, owes its name to its ability to adhere to human cells, thereby favouring the entry of Neisseriae. To circumvent the immune system, meningococci possess formidable machineries that allow them to secrete proteins in different manners; in particular, Neisseriae mainly use the autotransporter pathway (also known as type V secretion system) [216]. The first-described protein belonging to the autotransporter superfamily was an IgA protease from* N. gonorrhoeae* [217]. MspA (meningococcal serine protease A) is another putative autotransporter protein. Not all strains of* Neisseria gonorrhoeae*/*meningitidis* possess the gene for MspA/AusI (also known as NMB1998). However, Turner et al. [218] demonstrated that convalescent sera from subjects affected by serogroup B invasive disease recognized the MspA antigen. NhhA (*Neisseria* hia/hsf homologue A, also known as Hsf) and Neisserial adhesin A (NadA) also belong to the autotransporters. Nhha contributes to bacterial adhesion by binding heparin sulphate and laminin. In addition, through the activation of caspase, NhhA increases the rate of macrophage apoptosis [168, 219]. NadA, which is expressed by 50% of virulent strains [160], but only by 5% of the strains isolated from carriers, is of particular interest because it is one of the components of the 4CMenB (Bexsero) vaccine and binds beta1 integrins [220].

### 3.4. *N. meningitidis*: Avoidance Mechanisms against Reactive Oxygen Species (ROS), Reactive Nitrogen Species (RNS), and Antimicrobial Peptides (AMPs)

When in contact with the mucosa of the nasopharynx,* N. meningitidis* can implement several strategies of adhesion, but it must overcome many barriers of innate immunity. We have already mentioned how the capsule allows bacteria to mitigate the activity of DCs. However, it must elude other substances, such as the reactive oxygen species and reactive nitrogen species produced by macrophages and the antimicrobial peptides produced by activated neutrophils. As already mentioned, the capsule protects* N. meningitidis* from LL-37 cathelicidin, but LPS also contributes to the resistance of the bacterium against this cathelicidin [156].

Furthermore, the toxic action of ROS is neutralized by the secretion of enzymatic proteins, such as catalase and superoxide dismutase [144, 149, 221]. The gene that codes for catalase is katA and is regulated by OxyR [185, 186, 222], while SodB and SodC code for superoxide dismutase [223]. Laz, a lipid-modified azurin, protects the pathogen against H_2_O_2_ and cupper toxicity [135–138, 145, 176].

In addition,* N. meningitidis* possesses genes, which encode enzymes able to exert a denitrification action, such as aniA, CycP, nirK, nsrR, and norB. They favour the growth of the pathogen, enabling utilization and consumption of NO during microaerobic respiration, enhance pathogen survival, and protect* Neisseria* from nitrosative stress during colonization and invasion by preventing host cell S-nitrosothiol formation. Moreover, they reduce and downregulate the production of NO-dependent cytokines, such as TNF-alpha, IL-12, IL-10, CCL5 (RANTES), and CXCL8 (IL-8) [100, 101, 119, 120, 130, 157, 170, 172, 177, 224].

Contemporarily, already at the level of the mucosa, the microorganism must resist the complement system.

Another interesting mechanism is the strategy whereby* N. meningitidis* escapes the attempts of the host to sequester nutrients essential for growth and survival of the pathogen. This process has been termed “nutritional immunity” [131, 139]. The microbe is endowed with OM receptors (such as HmbR, HpuA or HpuB, TbpA or TbpB, and TdfF) for acquiring iron and other important metals [93, 124, 125, 128, 129, 140, 195, 225]. ZnuD is a high-affinity zinc uptake receptor, which plays an important role in enabling the pathogen to evade neutrophil-mediated killing [226, 227]. CbpA, a receptor for calprotectin, a protein released by neutrophils during inflammatory processes, is upregulated when* N. meningitidis* suffers from zinc limitation [226, 227]. Further examples of metabolic enzymes involved in nutritional immunity are glutamate transporters or molecules taking part in the carbon cycle [132–134, 228–230].

### 3.5. How Meningococcus Circumvents the Complement System

Three pathways can activate the complement functions, namely, the classic pathway, the alternative pathway, and the lectin pathway. All three of these pathways contribute to the transformation of C3 to C3b [231].

The alternative pathway acts by comparing self- with non-self-antigens and is activated by anything that differs from the markers of host cells. Specifically, factor H recognizes host-associated molecular patterns (HAMPs). Properdin, first identified in 1959, is another protein that can directly activate the alternative pathway of the complement system [232]. It has been demonstrated that properdin deficiency favours recurrent episodes of* N. meningitidis* infection [233].

Meningococci produce three different variants (1, 2, and 3) of a protein that binds factor H. This protein, named fHbp (factor H binding protein) or GNA1870, blocks activation of the alternative pathway of the complement system. Indeed, by surrounding themselves with fHbp,* N. meningitidis* cells capture and inactivate factor H. Thus, it is easy for the microorganism to survive and reproduce, especially in the bloodstream and cerebrospinal fluid. Hence, it is important that the 4CMenB (Bexsero) vaccine contains variant 1 of this protein, which is often expressed by virulent meningococcal strains [228]. Another vaccine (Trumenba), recently licensed in the USA, contains recombinant variants 1 and 2 of fHbp from* N. meningitidis* serogroup B, A and B subfamilies (A05 and B01, resp.) [234, 235]. The proteins are produced by exploiting an advanced genetic engineering technique, using* E. coli* as a vector.

### 3.6. The Adaptive Immune Response against* Neisseria meningitidis*

Microorganisms such as* N. meningitidis* are able to change many exposed surface proteins, while the polysaccharides, which constitute the capsule, are T-independent (TI-2) antigens and can activate B cells directly, without the intervention of the MHC. However, TI-2 antigens do not induce an efficient secondary response and do not induce the production of avid immunoglobulins. Rather, they induce the production of short-lived Abs belonging to the IgM class [236].

In addition, it is important to consider that, in infants and children, the development of the immune system is a dynamic process, which begins* in utero* and continues for months and even years after birth. This explains why many components of the immune system are inefficient or partially efficient in infancy and early childhood [237–239]. For this reason, most cases of meningitis and sepsis from* N. meningitidis* occur under the age of 4 years and particularly in the first year of life.

The critical role of bactericidal Abs against the exposed surface antigens of* N. meningitidis* has been demonstrated by several studies. Indeed, Goldschneider et al. [240] showed that only individuals without bactericidal Abs contracted the clinical disease. In addition, the successful therapeutic use of immune sera, which markedly reduced lethality when first implemented by Flexner [241], has shown the central role of these Abs in protecting against invasive disease. The opsonic activity of Abs is also very important in the protection against and the recovery from meningococcal disease, as is demonstrated by the role of neutrophils, macrophages, and DCs in combating* N. meningitidis*. It is also well known that the cerebrospinal fluid of patients contains large numbers of neutrophils full of microorganisms. These clusterings of neutrophils are known as neutrophil extracellular traps (NETs) and massively release cathepsin G.* N. meningitidis* circumvents these traps by blebbing spontaneously released OMVs (SOMVs). Other strategies that the pathogen exploits are modification of lipid A of LPS with phosphoethanolamine protected and upregulation of ZnuD [92].

The adaptive immune response has been studied in carriers and during both the invasive period leading to clinical meningitis and the convalescence period. T cell response is “two-faced”; while proinflammatory T cells may indeed blunt the invasive power of the pathogen, the induction of the Treg response, which is able to limit virulence, carries the price of the reduced effectiveness of the protective response, especially in children [242]. During infection, increased meningococcal antibody titres can be detected from the 4th day, peaking at the end of the third week or the beginning of the fourth week and showing a correlation with the severity of the disease and the age of the patient. In the acute period of the disease, the number of T cells generally drops, while that of B cells increases; by the end of the second week, IgG levels decline and IgM levels rise [243]. In particular, abnormalities in T cell response can be detected, such as an elevated percentage of CD25+ and HLA-DR+ T cells, an increase in CD4+ CD45R+ (suppressor-inducer) cells, with subsequent expression of activation antigens, and a decrease in CD4+ CDw29+ (helper-inducer) cells [244]. During convalescence, an age-associated Th response can be observed: specifically, a Th1 response (low IL-10/IFN-*γ* ratio) and a highly proliferative Th2 response (higher IL-10/IFN-*γ* ratio) can be detected in younger and older patients, respectively [245]. Generally, a significant CD4+ T central memory response, with serum bactericidal antibodies, a marker of protective immunity, can be found [246]. However, the above-mentioned age-related mucosal T effector/memory cell response may also be present without bactericidal antibodies [247].

### 3.7. Other Immunoescape Strategies

Temperature fluctuation plays an important, although underscored, role in microbial pathogenesis, colonization, invasion, and host evasion. In contrast to mammals that maintain constant body temperature, pathogens' and other animals' temperature oscillates on a daily basis. Loh and collaborators [99] have identified the molecular bases of this temperature-dependent strategy. They have studied three RNA thermosensors located in the 5′ untranslated regions (UTRs) of genes involved in the CP biosynthesis, the expression of fHbp, and sialylation of LOS/LPS. Increased temperature (e.g., during inflammation by coinfecting pathogens, such as influenza virus) “alarms” the meningococcus and triggers its defence mechanisms against human immune killing. This could be a key determinant for the transformation of a symbiont pathogen into a virulent one. However, the precise nature of this mechanism remains elusive.

Clustered regularly interspaced short palindromic repeats- (CRISPR-) Cas9 is another molecular tool that* Neisseria* can use in order to divert immune surveillance. It is an intrinsically ambivalent device, since, on one hand, being involved in gene expression and regulation, it restricts the possibility of editing the* Neisseria* genome via HGT/LGT or the insertion of exogenous nucleic material, and therefore it limits the microbial variability. On the other hand, CRISPR-Cas9-mediated repression of bacterial lipoprotein expression facilitates evasion of TLR2 by the pathogen [112].

Another mechanism is the molecular decoy, with which the microbe deceives the human immune system. For example,* fpr*B has an antigenic subdomain for binding antibodies, which is not essential for the functioning the autotransporter [94].

The example of* fpr*B is useful to show how* Neisseria* can use concurrently the previously described immunoescape strategies:* fpr*B is subject to a high degree of antigenic variation, is a phase-variable gene, is involved in nutritional immunity, and moreover exploits a molecular decoy. Neisserial carbohydrates, mimicking host carbohydrates, circumvent immune system and at the same time exploit their mimicry to recruit fH [91].

## 4. Meningococcal Vaccines

### 4.1. Polysaccharide Vaccines

As at least six meningococci are pathogenic in humans, the development of meningococcal vaccines has been a challenge. The first step towards solving the problem was to find a common denominator among antigens that showed high variability. In 1969, Gotschlich et al. [248] correctly put the first imperfect piece of the puzzle in place by demonstrating the possibility to extract the capsule polysaccharide. However, although high molecular weight groups A and C meningococcal polysaccharides proved immunogenic in adults [249–251], important limitations of this vaccine emerged in subsequent years. Indeed, while both group A and group C vaccines proved effective in USA and Italian recruits [252, 253] after one administration, they displayed only short-term efficacy in older children and adults. Moreover, vaccines against serogroup C did not prevent disease in infants, and the efficacy of group A vaccines in children under 1 year of age was unclear. The immune response occurred 10 days after vaccination. In schoolchildren and adults, one dose of these vaccines seemed to provide protection for at least 3 years [254]. These findings are explained by the characteristics of the antigens contained in the vaccines. Indeed, polysaccharides with repeating epitopes induce an immune response with the following characteristics [255, 256]:

- The response occurs between the ages of 3 and 18 months but is variable; in children less than 2 years of age, the response is usually poor;
- the affinity maturation of the Abs does not occur.
- The immunologic memory is not stimulated.
- Almost all (>90%) Abs produced belong to the IgM class and are produced in the spleen [257, 258]. Furthermore, several studies have suggested that vaccination with large amounts of polysaccharides induces immune tolerance towards these antigens [259].

### 4.2. Polysaccharide Conjugate Vaccines

Three immunogenic carrier proteins are generally used inpolysaccharide conjugate vaccines, namely, diphtheria or tetanus toxoid, CRM197 (a nontoxic variant of diphtheria toxin obtained by molecular biology), and a complex of outer membrane protein (OMP) mixture from* N. meningitidis*. The toxoids were selected as carriers, firstly because of their immunological potency and secondly because if the recipient had previously been immunized with the toxoid, a booster effect was predictable. Moreover, under particular conditions, suppressive effects can also occur. The conjugate vaccine against* N. meningitidis* serogroup C had great success in several countries (UK, Netherlands, Spain, Italy, etc.) [260]. Today, tetravalent conjugate vaccines ACWY are also available in both the USA and Europe [260–262].

### 4.3. Vaccines against* N. meningitidis* Serogroup B

The most difficult problem was to prepare a vaccine against* N. meningitidis* serogroup B strains. Indeed, the maximum expression of camouflage is found in the capsule of these strains. Specifically, the polysaccharide of serogroup B meningococcal strains is a homopolymer of sialic acid residues and has structural similarities to brain glycoproteins. For this reason, it was impossible to prepare a polysaccharide antimeningococcus B vaccine. To overcome this obstacle, OMVs-vaccines were developed and used during epidemics caused by* N. meningitidis* serogroup B strains. However, given the high variability of the proteins, such as porins, present in OMVs, these vaccines were effective only against very specific epidemic hypervirulent strains [263, 264]. In order to develop a vaccine against meningococcus B, other strategies were therefore implemented.

The most promising results were obtained through reverse vaccinology. This involves identifying the antigens for the vaccine not in the classic way—that is, from the components of the bacterium—but instead from the genes that express the proteins with the best characteristics to be good antigens for the vaccine. To obtain a universal vaccine against serogroup B meningococcal strains, complex bioinformatics software was also applied. Following the complete sequencing of the meningococcus B genome [265], researchers at Novartis Vaccines and Diagnostics identified 600 ORFs, which expressed proteins that are exposed on the surface of the bacterial cell. Subsequently, 350 proteins were successfully expressed in* E. coli*, purified, and used to immunize mice. Later, 28 novel protein antigens able to elicit Abs with bactericidal activity were identified. Finally, three of these 28 proteins were selected, namely, NHBA (GNA2132, fused with GNA1030 protein), fHbp (fused with GNA2091 protein), and* nad*A. NHBA (*Neisseria* heparin binding antigen), which is present in virtually all strains, binds heparin, which may increase the serum resistance of bacteria. fHbp (factor H binding protein) binds factor H, which enables the bacterium to survive in theblood [266, 267], thereby blocking the alternative pathway of the complement system.* nad*A (Neisserial adhesin A) promotes adherence to and invasion of human epithelial cells [161, 162]. In addition, the vaccine developed by Novartis Vaccines and Diagnostics contains a fourth component, namely, the vesicle of the OM from the New Zealand strains, which contain porin 1.4. Theoretically, this vaccine should elicit bactericidal Abs against the following:

- NHBA, thus increasing the bactericidal activity of the serum.
- fHbp, thus exposing* N. meningitidis* to the alternative pathway of the complement system.
- NadA, thus hindering the adherence of* N. meningitidis* to epithelial cells.
- Porin A 1.4 and other components of the mixture of proteins contained in OMVs; indeed, OMVs can enhance the immune response by functioning as a conjugate complex of proteins or, rather, a complex of adjuvants.

Several clinical trials have demonstrated the immunogenicity and safety of this vaccine, also named 4CMenB, in infants, children, adolescents, and adults [268, 269]. Consequently the vaccine has been approved by the EMA and named Bexsero [270]. It has also been approved by several national drug agencies (such as FDA), including the Agenzia Italiana del Farmaco (AIFA, Italian National Agency for Drugs) [271].

Owing to the wide variability of Neisserial antigens, a particular laboratory test (MATS) has been developed to estimate the potential effectiveness of the vaccine. Studies conducted worldwide have shown the potential effectiveness of Bexsero, which has been estimated at 87% in Italy [85].

Trumenba was approved for individuals aged 10 to 25 years in the USA in October 2014 [85]. The potential of the vaccine antigen was tested in the laboratory [272] and on a murine model [273, 274]. The immunogenicity, safety, and tolerability of this vaccine were investigated in a randomized controlled trial in infants aged 18–36 months, who were subdivided into three cohorts (receiving 20-, 60-, and 200-*μ*g rLP2086 dose, resp.) and matched with two control groups: one vaccinated against hepatitis A virus (HAV) and the other with a saline placebo. After the vaccination cycle, seroconversions against Neisserial strains expressing LP2086 variants homologous to the vaccine antigens were found in 61.1–88.9% of toddlers and against strains expressing heterologous LP2086 variants in 11.1–44.4%. Adverse reaction rates were negligible and the vaccine proved to be safe and well tolerated [275]. However, another randomized phase 1/2 clinical study found high fever rates in toddlers receiving one 20- or 60-*μ*g rLP2086 dose (64% and 90%, resp.) [276].

In a randomized study performed in the USA and Europe in a sample of adolescents (11–18 years of age), Trumenba proved to be highly immunogenic. The proportion of vaccinees with human serum bactericidal activity (h-SBA) titres with a ≥4-fold rise against hypervirulent Neisserial strains with different variants of fHbp was in the range of 75–100%. In another randomized clinical study, carried out in Australia, the safety and immunogenicity of the vaccine were assessed in 60 healthy adults (18–40 years of age) who received 120 *μ*g doses at 0, 1, and 6 months. The percentage of seroprotected vaccinees was 94.3% against the homologous strains and 70–94.7% against the heterologous strains. The vaccine was well tolerated [277, 278].

As fHbp is also expressed by Neisserial serogroups other than B, the anti-fHbp Abs elicited by rLP2086 might exert a bactericidal effect on meningococci, such as those against* N. meningitidis* serogroup C, as proved by Harris and collaborators [279] and by Konar and colleagues [280]. Moreover, there is some evidence that Trumenba could, at least in part, have an effect on carriage and reduce the risk of acquiring some hypervirulent strains [281].

### 4.4. New Vaccines

The currently available* Neisseria* vaccines, described in the previous paragraphs, are reported and summarized in Table 3.

The elucidation of immunoescape strategies and genomics have enabled scholars to discover new potential vaccine candidates, like NMB0928 [282] or NMB1468 [283], FrpB/fetA [125], LbpA and LbpB [284], adhesin complex protein (ACP) [285], NspA [286, 287], MIP [152], ZnuD [93], PilE [76] and PilQ [288], IgA protease [289], T cell stimulating protein A (*tsp*A) [289], or the CP polymerase of* Neisseria* serogroup X [290], among others [291].

Reverse vaccinology has proved to be a promising approach, enabling researchers to develop the effective vaccine Bexsero. New highly integrated approaches, which combine genomics with postgenomics, are leading to next-generation vaccines. A combination of reverse and forward vaccinology techniques, such as immunoproteome investigation via combined cell surface immunoprecipitation and mass spectrometry (MS) [153], and new bioinformatics strategies, such as the protectome approach [292], are promising in identifying highly conserved motifs in known bacterial protective antigens and using them for the design of effective universal vaccines [293, 294].

## 5. Conclusions

The development of effective vaccines against meningococcal disease has been a long and hard struggle. Early efforts yielded only partial results, with the creation of polysaccharide vaccines [295]. Subsequent research, however, led to the production of the conjugate vaccines [296]. Today, we have the conjugate meningococcal C vaccines [297], an affordable conjugate vaccine against* N. meningitidis* serogroup A (MenAfriVac) [298, 299], the tetravalent conjugate vaccines [270], and, finally, two “universal” vaccines against meningococcal group B strains [300, 301]. The critical rate of coverage required in order to eliminate the disease is probably not among the highest [302]. Indeed, the conjugate vaccine for serogroup C has resulted in dramatic reductions of cases of the disease [303] and created herd immunity that seems to have had a significant effect on the carrier status of adolescents and young adults. Thus, the prospect of dominating this very serious disease lies decidedly in the medium term.

However, it must be borne in mind that we are immersed in a constellation of* Neisseriae*, whose only survival niche is man. Although Neisseriae such as* N. lactamica*,* N. sicca*,* N. elongata*,* N. cinerea*, and* N. flavescens* are usually able to establish silent infection in normal humans, it is not inconceivable that, given the microorganism's great capacity for genetic variation, nonpathogenic* Neisseria* might become hazardous to humans [304].

The challenge is still open.

## Figures and Tables

**Table 1:** An overview of the most important immunoescape strategies exploited by *Neisseria meningitidis*.

Immunoescape mechanism	Details	References
Structural/antigenic variation	It consists in the modified expression of domains, which are antigenically different within a clonal population, by which the pathogen is able to escape the host immunity selection and circumvent the immune surveillance It usually involves LOS/LPS, opacity, and pilin proteins LOS/LPS and opacity factor structural/antigenic variation depends essentially on phase variation Pili antigenic variations depend on RecA-mediated recombination	[31, 75, 76]

Autolysis	It is mediated by OMPLA	[77]

Blebbing and microvesicles formation	The blebs originate as evaginations of the outer layer	[78]

Capsule switching	Due to microevolution, there is shift from serogroup B to serogroup C, from serogroup C to W-135, from serogroup Y to W-135, and from serogroup Y to B; nanostructured materials such as MWNTs and mesoporous silica increase transformational capacity	[30, 79–87]

Capsule modification	For example, modification of lipid A of meningococcal LOS/LPS with phosphoethanolamine protects *Neisseria* from neutrophils-mediated killing Another example is given by the O-acetylation of capsular antigens (LpxL2 gene mutants are indeed more virulent) LpxL1 gene mutants activate TLR4 less efficiently	[88]

Genome plasticity	HGT/LGT (via conjugation, transduction, and transformation) and homologous intragenic recombination	[25, 27, 30, 89]

Host modification	*Neisseria* exploits a bacterial sialyltransferase scavenging available host CMP-NANA for modifying LOS/LPS	[70]

Molecular mimicry	CP of serogroup B strain is a homopolymer of *α*2-8-linked sialic acid and is similar to NCAM-1 L-NNT in the lipopolysaccharide of virulent strains is similar to an antigen on red blood cells DMP19 acts as a DNA-mimic protein	[67, 69, 71–74, 90, 91]

Metabolic pathways	Examples are iron, lactate, glutamate uptake, utilization, and avoidance of neutrophil oxidation burst, ROS, and RNS	[92, 93]

Molecular decoy	FprB has an antigenic subdomain for binding antibodies, which is not essential for the functioning of the autotransporter; it also blebs with OMPs and LPS/LOS distract the immune system, directing the response away from the microbe	[94]

Immunotype switch	LPS immunotype switches from L3 to L8/L1 by lgtA, lgtC phase variation LOS immunotype can contribute to immunoescape	[95, 96]

Phages and prophages	The pathogen hosts a number of prophages, from the Mu-related family to the phage l-related group and the family of filamentous M13-like phages	[25, 30, 89]

Phase variation	High-frequency reversible changes can occur in the length of SSRs (of capsule, LOS, opacity factor, porin, adhesin, invasin, autotransporter, haemoglobin receptor, DNA mismatch repair, and pilin genes, termed as contingency genes and organized in modules called phasevarions) Other repeat sequences can be REP2, CRs, CREEs, and NIMEs Transposon-like elements can play a role Phase variation mediates resistance to antibiotics Phase variation mediates carriage persistence	[50, 52, 59]

Pilin conversion and modification	Pilin is posttranslationally modified by addition of a glycan, two phosphorylcholines, and a glyceramido acetamido trideoxyhexose residue	[97, 98]

Plasmid	Examples of plasmids that can contribute to *Neisseria* variability are pJS-A, pJS-B	[33]

Recruitment of human components of immune system	*Neisseria* escapes complement-mediated killing recruiting and sequestering fH to its surface	[91]

Temperature-regulated defence	RNA thermosensors finely tune the expression of CP components, LOS, and fHBP, thus protecting against human immune killing	[99]

CMP-NANA: cytidine 5′-monophospho-N-acetylneuraminic acid; CP: capsule; CRs: Correia repeats; CREE: Correia repeat-enclosed element; DNA: deoxyribonucleic acid; fH: complement factor H; fHBP: fH binding protein; HGT: horizontal gene transfer; lgt: prolipoprotein diacylglyceryl transferase; L-NNT: lacto-N-neotetraose; LOS: lipooligosaccharide; LPS: lipopolysaccharide; LGT: lateral gene transfer; MWNTs: multiwalled nanotubes; NCAM-1: neural cell adhesion molecule 1; NIME: Neisserial intergenic mosaic element; OMPs: outer membrane proteins; OMPLA: outer membrane phospholipase A; RecA: recombinase A; REP2: repetitive extragenic palindromic sequence; RNA: ribonucleic acid; RNS: reactive nitrogen species; ROS: reactive oxygen species; SSRs: simple sequence repeats; TLR: toll-like receptor.

**Table 2:** An overview of the most important genes and gene products of *Neisseria meningitidis* involved in immunoescape mechanisms.

*N. meningitidis* molecule	Immunological role	Reference
*ani*A	A nitrite reductase: it protects *Neisseria* from nitrosative stress during both colonization and invasion	[90, 100–102]

*App *	It is phase-variable	[103]

*ausI/*MspA	An autotransporter and a serine protease; it is phase-variable	[45, 46]

Biofilm (and molecules involved in the biofilm synthesis, such as *aut*A or *hrp*A, or optimizing pathogen survival in biofilm, such as the alpha-peptide of IgA1 protease, *adh*C, *est*D)	Biofilm protects from macrophages; *adh*C is involved in S-nitrosoglutathione metabolism and in glutathione-dependent detoxification system; EstD is involved also in *Neisseria* colonization	[104–108]

Blebs (with OMPs and LPS/LOS) and SOMVs	They protect from neutrophils-mediated killing and NETs; they divert the immune response away from the pathogen	[78]

Capsule and molecules involved in the capsule synthesis such as *kps*C, *kps*S	It activates TLR2 pathway, it increases serum resistance, and it inhibits the classical pathway of complement	[109–111]

Cas9 and the CRISPR-Cas system	CRISPR-Cas9-mediated repression of bacterial lipoprotein expression facilitates evasion of TLR2 by the pathogen; it is involved in gene expression and regulation	[112, 113]

*cbp*A	It mediates zinc piracy and protects from nutritional immunity	[93]

*Cps *	As a gene, it is involved in the capsule biosynthesis; as RNA, it acts as a thermosensor; Cps gene amplification protects the pathogen	[99, 114]

*CrgA *	It is involved in the regulation of pili and capsule expression; it plays a major role in the infectious cycle of *Neisseria *	[114–116]

*Css *	As a gene, it is involved in the capsule biosynthesis; as RNA, it acts as a thermosensor	[99]

*ctr*A, *ctr*D	As genes, they are involved in the capsule export; as RNAs, they act as thermosensors; IS1301 in the IGR between *sia* and *ctr* operons mediates resistance to Abs	[99, 117, 118]

*cyc*P	It is involved in denitrification metabolism and protects *Neisseria* from nitrosative stress	[90, 119, 120]

*dam *	It is involved in phase variation and modulation	[42]

*dca*C	It is phase-variable	[40]

*din*B	A DNA polymerase IV belonging to the SOS regulon: it is involved in phase variation and modulation	[42]

DNA mismatch repair genes (*fpg*, *mut*L, *mut*S, *mut*Y, *rec*A, *rec*N, *uvr*D)	They are phase-variables; they protect against oxidative stress	[42, 48, 51, 121]

*drg *	It is involved in phase variation and modulation	[42]

*far*A, *far*B, *far*R	They remove antimicrobial peptides, proteases, lysozyme, and acids from the bacterial cytosol and protect the pathogen	[122, 123]

*fbp*A, *fbp*B	They are involved in phase variation and modulation	[51]

*Feta *	It is involved in phase variation and modulation	[124–126]

fHbp (formerly known as GNA1870)	It is involved in phase variation and modulation; it protects *Neisseria* from complement-mediated killing, binding fH	[90, 127]

*frp*A, *frp*B, *frp*C	They are phase-variable; they can act as a molecular decoy	[124, 125, 128]

*fun*Z	It is a site of bacteriophage insertion; it is phase-variable	[49]

*fur *	It is involved in phase variation and modulation; it tunes the gene expression of virulence genes	[102, 129]

*ggt *	It regulates pathogen growth	[130]

*Ght *	It is involved in the capsule biosynthesis and in the resistance mechanisms of the pathogen	[131, 132]

*glt*T	It favours meningococcal internalization into human endothelial and epithelial cells; it regulates pathogen growth	[133, 134]

H.8	AAEAP motifs are target for generation of blocking Abs	[135–138]

Haemoglobin-linked iron receptors (*hpuA*, *hpuB*, *hmbR*)	They are involved in phase variation and modulation	[43, 139–141]

*Hfq *	A RNA chaperone: it is involved in stress response and virulence and is a pleiotropic regulator of protein expression	[142]

*hsd*S	It is phase-variable	[49]

IgA protease	It cleaves secretory IgA, hinders Ab binding and function, and may play role in biofilm formation; it cleaves lysosomal LAMP1 in epithelial cells; moreover, it is phase-variable	[122, 142, 143]

*kat*A	It confers resistance to RNS, including peroxynitrite (PN), protects against ROS, and detoxifies H_2_O_2_	[90, 102, 122, 144]

Laz	A lipid-modified azurin: it protects against hydrogen peroxide and copper toxicity; it promotes *Neisseria* growth and survival	[135, 138, 145]

*lbpA*, *lbpB *	They are involved in iron acquisition and metabolism; they are phase-variable; moreover, the release of LbpB enables *Neisseria* to escape from complement-mediated killing	[90, 122, 146]

*lct*P	Its inactivation results in C3-mediated cell lysis	[102, 147, 148]

*lgt*A, *lgt*B, *lgt*C, *lgt*D, *lgt*E, *lgt*G	They are involved in LOS biosynthesis and are phase-variable; for example, *lgt*A or *lgt*C phase variation mediates LPS immunotype switch from L3 to L8/L1	[60]

LOS/LPS	It protects from macrophages; strains of the same species produce different LOS glycoforms	[122]

*lpt*A	It adds a phosphoethanolamine group to lipid A and confers resistance to defensins and cathelicidins	[90, 149]

*Lst *	LOS sialylation (by the enzyme Lst) prevents complement deposition and phagocytosis by neutrophils	[122, 150]

*mes*J	It is phase-variable	[49]

*Msf *	It binds to vitronectin; it increases serum resistance	[151]

Mip	It tunes gene expression	[102, 152, 153]

*mis*R, *mis*S	They are phase-variable; they are involved in capsule regulation and modification	[114, 154]

*mlt*A (formerly known as GNA33)	It tunes gene expression	[155]

*mnt*A, *mn*tB, *mnt*C	They protect against oxidative stress	[122, 156]

*mod*A, *mod*B	They are phase-variable	[34]

*msr*A, *msr*B	They are involved in the methionine sulfoxide reduction and they repair oxidized proteins	[122, 157]

*mtr*C, *mtr*D, *mtr*E	They protect against cationic antimicrobial peptides and toxic hydrophobic molecules	[122, 158, 159]

*nadA* and its regulator *nad*R	It binds to Hsp90, recruits ARF6 and Rab11, and activates human monocytes and macrophages, triggering IFN-gamma and R-848 dependent pathways; it interacts with beta1 integrins; it is phase-variable	[39, 160–165]

*nal*P	An autotransporter protease: it cleaves C3, facilitates degradation of C3b, and enhances Neisserial survival in human serum; it stabilizes the biofilm; moreover, it is involved in the processing of other proteases, such as the proteases which release LbpB, whose release enables *Neisseria* to escape from complement-mediated killing; NalP processes also App and IgA1 protease; it has an important role in the virulence of the pathogen	[24, 102, 166]

Nhba (formerly known as GNA2132)	It tunes gene expression	[167]

*nhhA *	It activates TLR4-dependent and independent pathways; it triggers apoptosis in macrophages; it increases serum resistance and protects from phagocytosis and complement attack; it is essential for colonization	[168, 169]

*nif*S	It is phase-variable	[49]

*nir*K	It protects *Neisseria* from nitrosative stress during colonization and invasion	[170, 171]

*nor*B	It favours the pathogen growth, enabling utilization and consumption of NO during microaerobic respiration, enhances pathogen survival, protects *Neisseria* from nitrosative stress during colonization and invasion, decreases and downregulates the production of NO-regulated cytokines, such as TNF-alpha, IL-12, IL-10, CCL5 (RANTES), and CXCL8 (IL-8), and prevents host cell S-nitrosothiol formation	[100, 119, 120, 170, 172]

*nsp*A	It binds to factor H and inhibits AP	[122, 173–175]

*nsr*R	It is involved in denitrification metabolism and protects *Neisseria* against nitrosative stress	[176, 177]

*oat*W, *oat*Y	They tune gene expression	[178]

Opa	It interacts with CEACAM, promoting endothelial cell attachment and upregulating endoglin (CD105) and cooperation with *β*1 integrins; it elicits innate host defences and actively suppresses adaptive immune responses that would eliminate the pathogen	[179–184]

Opc	It binds to vitronectin, it inhibits AP, and it increases serum resistance; it elicits innate host defences and actively suppresses adaptive immune responses that would eliminate the pathogen	[179–182, 184]

*oxy*R	It regulates catalase expression and is involved in the protection from oxidative stress	[185, 186]

P36	It is involved in Neisserial adhesion.	[187]

*pac*A, *pac*B	They are involved in the composition and regulation of peptidoglycan membrane	[188]

*pgl*A, *pgl*B, *pgl*G, *pg*lH	They are phase-variable	[60–62]

Pili	They alter the expression levels of human genes known to regulate apoptosis, cell proliferation, inflammatory response, adhesion, and genes for signaling pathway proteins such as TGF-beta/Smad, Wnt/beta-catenin, and Notch/Jagged	[189]

*pil*C1	It interacts with mucosal surface and mediates the crossing of the BBB	[41, 169]

P*ilE*, *pil*S	They are involved in nonreciprocal recombination-based antigenic variation	[76]

P*ilE*, *pil*V	They bind to CD147 for vascular colonization; they mediate also *Neisseria* internalization	[190, 191]

*pil*P, *pil*Q	They are involved in pilus biogenesis and outer membrane stabilization	[51, 192]

*porA *	It binds to fH, C3b, C4b, and C4bp (more strongly under hypotonic conditions); it increases serum resistance; it is involved in phase variation	[122, 139, 173]

*por*B	It inhibits factor H-dependent AP; it interacts with TLR1 and TLR2 and activates I*κ*B, MAPK/MAPKK, and PTK pathways, leading to CD86 upregulation, to IL-6, IL-12, and TNF secretion in B cells and DCs, and to IgB secretion in B cells	[122, 173]

*ppt*A	It is phase-variable	[47]

*Ppx *	It is an exopolyphosphatase whose mutation protects *Neisseria* from complement-mediated killing; it interacts with the AP of the complement activation	[64]

*rmp*M	It is involved in phase variation and modulation	[193, 194]

Sialic acid synthase (*neu*B, *sia*A, *sia*B, *sia*C, *syn*C)	They are phase-variable	[102]

*sod*B, *sod*C	They protect from phagocytosis by human monocytes/macrophages	[102]

*tbpA*, *tbpB* (also known as *tbp*1, *tbp*2)	They are involved in nutritional immunity	[121]

*TdfF *	It is involved in intracellular iron acquisition and is found only in genomes of pathogen strains	[28]

Temperature sensors (such as RNA thermosensors located in the 5′ UTRs of genes necessary for capsule biosynthesis, the expression of fHbp, and sialylation of LOS/LPS)	Activated by coinfecting pathogens, they recruit mechanisms of resistance and immunity escape	[99]

*ton*B	It is involved in nutritional immunity, supplying energy to the pathogen	[93]

Uncharacterized proteins (NGO1686, NMB0741, NMB1436, NMB1437, NMB1438, and NMB1828)	They protect from nonoxidative factors, but their mechanisms are still not understood; NMB1436, NMB1437, and NMB1438 are putatively involved in iron metabolism	[122, 195]

Uncharacterized factor (NMA1233)	It is involved in phase variation and modulation	[26, 51]

*xse*B	It is involved in phase variation	[26]

*znuD *	It protects from neutrophils and nutritional immunity	[92]

Ab: antibody; AP: Alternative Pathway; ARF6: ADP-ribosylation factor 6; App: adhesion and penetration protein; BBB: blood-brain barrier; cbp: calprotectin binding protein; CEACAMs: carcinoembryonic antigen-related cell adhesion molecules; CRISPR: clustered regularly interspaced short palindromic repeats; ctr: capsule transport apparatus; dam: DNA adenine methyltransferase; drg: dam replacing gene; fur: ferric uptake regulator; ggt: gamma-glutamyl aminopeptidase; hsp: heat-shock protein; IgA: immunoglobulin A; lbp: lactoferrin binding protein; lct: lactate permease; LOS: lipooligosaccharide; Mip: macrophage infectivity potentiator; mltA: membrane-bound lytic transglycosylase A; IGR: intergenic region; Msf: meningococcal surface fibril; Msr: methionine sulfoxide reductase; NadA: *Neisseria* adhesion A; NhhA: *Neisseria* hia homologue A; oat: O-acetyltransferase; OMV: outer membrane vesicle; opa: opacity-associated protein a; opc: opacity-associated protein c; pac: peptidoglycan O-acyltransferase; pil: pilin; por: porin; RNA: ribonucleic acid; RNS: reactive nitrogen species; Sod: superoxide dismutase; SOMVs: spontaneously released OMVs; Tbp: transferrin-binding protein; TLR: toll-like receptor; UTRs: untranslated regions; uvr: ultraviolet resistant.

**Table 3:** An overview of the currently available *Neisseria meningitidis* vaccines.

Vaccine	Manufacturer	Serogroups	Licensed age group	Administration schedule	Components details
AC Vax	GlaxoSmithKline, UK	A, C	2 y+	Single dose	50 *μ*g each of meningococcal polysaccharides

ACWY Vax	GlaxoSmithKline, UK	A/C/Y/W-135	2 y+; can be given also at 2 mo+, even though less protective against C, Y, and W-135	Single dose	50 *μ*g each of meningococcal polysaccharides

Bexsero (4CMenB)	Novartis Vaccines and Diagnostics	B	2 mo–17 y	Complex dose schedule depending on age: 3 doses + booster for 2–5 mo; 2 doses + booster at 6–23 mo; 2 doses at 2+ y	50 *μ*g of each recombinant NHBA, NadA, fHbp fusion proteins, OMVs from strain NZ98/254 containing the PorA P1.4 (25 *µ*g), adsorbed on 0.5 mg Al^3+^

HexaMen and HexaMix	National Institute for Public Health and the Environment, Bilthoven, Netherlands	B	—	2, 3, and 4 mo, a booster dose at 12–18 mo	OMV from two recombinant engineered strains, each of which expressed three different PorA subtypes (P1.5-2, 10; P1.12-1, 13; P1.7-2, 4; P1.19, 15-1; P1.7, 16; and P1.5-1, 2-2)

Menactra (MenACWY-DT)	Sanofi Pasteur	A/C/Y/W-135	9 mo–55 y	Single dose	4 *μ*g each of meningococcal polysaccharides conjugated to 48 *μ*g of a diphtheria toxoid protein carrier

MenAfriVac (MenA-TT)	Serum Institute of India	A	1–29 y	Single dose	10 *μ*g of meningococcal group A polysaccharides conjugated to 10 to 33 *μ*g tetanus toxoid

MenBvac	National Institute for Public Health, Norway, and Novartis	B	—	3 doses (interval 5–12 w)	OMVs from the strain 44/76 adsorbed on Al^3+^

MencevaxA	GlaxoSmithKline and RIT, Belgium	A	2 y+	Single dose	50 *μ*g of meningococcal group A polysaccharides No conjugation

MencevaxAC	GlaxoSmithKline	A, C	2 y+	Single dose	50 *μ*g each of meningococcal group polysaccharides No conjugation

MencevaxACY	GlaxoSmithKline	A, C, Y	2 y+	Single dose	50 *μ*g each of meningococcal group polysaccharides No conjugation

MencevaxACYW	GlaxoSmithKline	A/C/Y/W-135	2 y+	Single dose	50 *μ*g each of meningococcal group polysaccharides No conjugation

Mengivac A + C (MenPS)	Sanofi Pasteur	A, C	—	—	50 *μ*g of meningococcal group C polysaccharides

MenHibrix (HibMenCY-TT)	GlaxoSmithKline	C, Y	6 w–18 mo	2, 4, 6, and 12 to 15 mo	Meningococcal groups C and Y polysaccharides conjugated to tetanus toxoid

Meningitec (MenC-CRM)	Wyeth Vaccines, Canada, UK, and Australia	C	2 mo+	3 doses at 2–12 mo, 1 dose at 12 mo+	10 *μ*g of meningococcal group C polysaccharides conjugated to 15 *μ*g CRM_197_

Meninvact	Sanofi Pasteur	C	2 mo+	2 doses at 2–12 mo, 1 dose at 12 mo+	Meningococcal group C polysaccharides conjugated to CRM_197_

Menitorix (Hib-MenC-TT)	GlaxoSmithKline	C	6 w–12 mo	Booster at 1-2 y	Meningococcal group C polysaccharides conjugated to tetanus toxoid

Menjugate (MenC-CRM)	Novartis Vaccines and Diagnostics	C	2 mo+	3 doses at 2–12 mo; 1 dose at 12 mo+	10 *μ*g of meningococcal group C polysaccharides conjugated to 12.5 to 25 *μ*g CRM_197_

Menomune	Sanofi Pasteur	A, C	2 y+	Single dose	50 *μ*g each of meningococcal group polysaccharides No conjugation

Menomune	Sanofi Pasteur	A/C/Y/W-135	2 y+	Single dose	50 *μ*g each of meningococcal group polysaccharides No conjugation

Menovac	Finlay Institute	A/C/Y/W-135	2–55 y	Single dose	Meningococcal group polysaccharides

Menveo (MenACWY-CRM197)	Novartis Vaccines and Diagnostics	A/C/Y/W-135	2–55 y	Single dose	10 *μ*g of meningococcal group A polysaccharides and 5 *μ*g of meningococcal groups C, Y, and W-135 polysaccharides conjugated to CRM_197_

MeNZB	Institute for Public Health, New Zealand, Chiron, Novartis	B	—	—	OMVs from strain P1.7b, 4

NeisVac-C (MenC-TT)	Baxter BioScience	C	2 mo–65 y	2 doses at 2–12 mo (with an interval of at least 2 mo), 1 dose at 12 mo+	10 *μ*g of meningococcal group C polysaccharides conjugated to tetanus toxoid

Nimenrix	GlaxoSmithKline	A/C/Y/W-135	1 y+	Single dose	5 *μ*g each of meningococcal group polysaccharides conjugated to 44 *μ*g tetanus toxoid

NmVac4	JN-International Medical Corporation	A/C/Y/W-135	2–55 y	Single dose	50 *μ*g each of meningococcal group polysaccharides

Trumenba	Pfizer	B	10–25 y	3 doses (0–2–6 mo)	120 *µ*g of recombinant fHbp adsorbed on 0.25 mg Al^3+^

Zamevax	Imunoloski Zavod, Croatia	A, C	—	—	No conjugation

CRM_197_: cross-reacting material 197; fHbp: factor H binding protein; mo: month; NadA: *Neisseria* adhesion A; NHBA: Neisseria heparin binding antigen, also named GNA2132; OMV: outer membrane vesicle; PorA: porin A; w: week; y: year; Al^3+^: Aluminum.

## Abbreviations

Abs: Antibodies
ACP: Adhesin complex protein
AIFA: Agenzia Italiana del Farmaco (Italian National Agency for Drugs)
AMPs: Antimicrobial peptides
AP: Alternative Pathway
*app*: Adhesion and penetration protein
ARF6: ADP-ribosylation factor 6
BBB: Blood-brain barrier
C3: Human complement 3 component
C3b: Human complement 3b component
C4: Human complement 4
C4bp: Human complement 4b binding protein
CAMPs: Cationic antimicrobial peptides
*cas*: CRISPR-associated
*cbp*: Calprotectin binding protein
CCL: Chemokine (C-C motif) ligand
CD: Cluster of Differentiation (CD4, CD25, CD45R, CD86, CD105, CD147, CDw29)
CDS: Coding sequence
CP: Capsule
CEACAMs: Carcinoembryonic antigen related cell adhesion molecules
CMP-NANA: Cytidine monophosphoacetyl* N*-Acetylneuraminic acid
CRs: Correia repeats
CREEs: Correia repeat-enclosed elements
CRISPR: Clustered regularly interspaced short palindromic repeats
CRM197: Cross-reacting material 197
CRP: C-reactive protein
CXCL: Chemokine (C-X-C motif) ligand
DCs: Dendritic cells
*dam*: DNA adenine methyltransferase
DNA: Deoxyribonucleic acid
*drg*: Dam replacing gene
dRS3: Duplicated repeat sequence 3
*E. coli*: * Escherichia coli*
EMA: European Medicines Agency
FDA: Food and Drug Administration
fH: Factor H
fHbp: Factor H binding protein
*fur*: Ferric uptake regulator
GEI: Genomic island
GGI: Gonococcal genetic island
*ggt*: Gamma-glutamyl aminopeptidase
GNA: Genome-derived neisserial antigen (GNA33, GNA1030, GNA1870, GNA2091, GNA2132)
GWAS: Genome-wide association studies
HAMPs: Host-associated molecular patterns
HAV: Hepatitis A virus
HGT: Horizontal Gene Transfer
HLA: Human leukocyte antigens
*hpu*: Haemoglobin-haptoglobin utilization
h-SBA: Human serum bactericidal activity
HSP: Heat-Shock Protein
HSPG: Heparan sulphate proteoglycan
HTTs: High-throughput technologies
IFN: Interferon
IgA: Immunoglobulin A
IgG: Immunoglobulin G
IgM: Immunoglobulin M
IGR: Intergenic region
I*κ*B: Inhibitors of NF-*κ*B
IL: Interleukin (IL6, IL8, IL10, IL12)
IM: Inner membrane
IS: Insertion sequence (IS1016, IS1106-like, IS1301, IS1655)
*kat*A: Catalase A
LAMP1: Lysosomal-associated membrane protein 1
*lbp*: Lactoferrin-binding protein
*lct*: Lactate permease
LGT: Lateral Gene Transfer
*lgt*: Prolipoprotein diacylglyceryl transferase (*lgt*A,* lgt*B,* lgt*C,* lgt*D,* lgt*E,* lgt*G)
L-NNT: Lacto-N-neotetraose
LOS: Lipooligosaccharides
LPS: Lipopolysaccharides
MAC: Membrane attack complex
MAPK: Mitogen-activated protein kinase
MAPKK: Mitogen-activated protein kinase Kinase
MATS: Meningococcal Antigen Typing System
MDA: Meningococcal disease-associated island
MHC: Major histocompatibility complex
MIP: Macrophage infectivity potentiator
MMEs: Minimal mobile elements
*mlt*A: Membrane-boundlytic transglycosylase A
mo: Month
mRNA: messenger RNA
*msf*: Meningococcal surface fibril
*msr*: Methionine sulfoxide reductase
MS: Mass spectrometry
*msp*A: Meningococcal serine protease A
*mtr*: Multiple transferable resistance
MWNTs: Multiwalled carbon nanotubes
*N.*: Neisseria (*N. lactamica*,* N. sicca*,* N. elongata*,* N. cinerea*, and* N. flavescens*)
*nad*A: Neisserial adhesin A
NCAM-1: Neural cell adhesion molecule 1
NEMIS: * Neisseria* miniature insertion sequences
NETs: Neutrophil extracellular traps
NF-*κ*B: Nuclear factor kappa-light-chain-enhancer of activated B cells
*N. gonorrheae*: * Neisseria gonorrheae*
NHBA: Neisserial heparin binding antigen
*nhhA*: * Neisseria* hia/hsf homologue
NIMEs: Neisserial intergenic mosaic elements
NKs: Natural killer cells
NLRs: NOD-like receptors
*N. meningitidis*: * Neisseria meningitidis*
NO: Nitric oxide
NOD: Nucleotide-binding Oligomerization Domain
NTPase: Nucleoside triphosphatases
*oat*: O-acetyltransferase
OM: Outer membrane
OMPs: Outer membrane proteins
OMVs: Outer membrane vesicles
OMPLA: Outermembrane phospholipase A
Opa: Opacity-associated protein a
Opc: Opacity-associated protein c
ORF: Open reading frame
*pac*: Peptidoglycan O-acyltransferase
PAI: Pathogenic island
PAMPs: Pathogen-associated molecular patterns
*pil*: Pilin (*pil*C: OM/cell surface pilin;* pil*D: prepilin-processing leader peptidase;* pil*F: traffic NTPase;* pil*G: pilus assembly protein;* pil*M: biogenesis protein;* pil*P: pilot protein;* pil*Q: secretin;* pil*T: traffic NTPase;* pil*W: pilus stabilization protein)
PN: Peroxynitrite
*por*: Porin
*ppt*A: Pilin phosphorylcholine transferase A
PRRs: Pattern recognition receptors
PTK: Protein tyrosine kinase
RANTES: Regulated on activation, normal T cell expressed and secreted
RecA: Recombinase A
REP2: Repetitive extragenic palindromic sequence
RIG-I: Retinoic acid-inducible gene 1
rLP2086: Recombinant lipoprotein 2086
RLRs: RIG-1-like receptors
RNA: Ribonucleic acid
RNS: Reactive nitrogen species
ROS: Reactive oxygen species
*sia*: Polysialic acid capsule biosynthesis protein (*sia*A,* sia*B,* sia*C,* sia*D)
SMAD: Small Mothers Against Decapentaplegic
*sod*: Superoxide dismutase (sodB, sodC)
SOMVs: Spontaneously released OMVs
SSM: Slipped strand mispairing
SSR: Simple Sequence Repeat
*tbp*: Transferrin-binding protein
TGF: Transforming Growth Factor
Th: T-cell helper
TI: T-cell Independent
TLR: Toll-like receptor (TLR2, TLR4, TLR5, TLR9)
TNF: Tumor Necrosis Factor
*tsp*A: T cell stimulating protein A
UK: United Kingdom
USA: United States of America
UTR: Untranslated Region
w: Week
WGS: Whole-genome sequencing
WNT: Wingless-related integration site
y: Year
*znu*D: Zinc uptake component D.
